# Detection of Shiga Toxin 2 Produced by *Escherichia coli* in Foods Using a Novel AlphaLISA

**DOI:** 10.3390/toxins10110422

**Published:** 2018-10-23

**Authors:** Cheryl M. Armstrong, Leah E. Ruth, Joseph A. Capobianco, Terence P. Strobaugh, Fernando M. Rubio, Andrew G. Gehring

**Affiliations:** 1 Molecular Characterization of Foodborne Pathogens Research Unit, United States Department of Agriculture, Eastern Regional Research Center, Wyndmoor, PA 19038, USA; Cheryl.Armstrong@ars.usda.gov (C.M.A.); Joseph.Capobianco@ars.usda.gov (J.A.C.); Terence.Strobaugh@ars.usda.gov (T.P.S.J.); 2 Abraxis, Inc., Warminster, PA 18974, USA; lruth@abraxiskits.com (L.E.R.); frubio@abraxiskits.com (F.M.R.)

**Keywords:** amplified luminescent proximity homogenous assay-linked immunosorbent assay (AlphaLISA), detection, enzyme-linked immunosorbent assay (ELISA), *E. coli*, Shiga toxin, STEC, Stx2

## Abstract

Amplified luminescent proximity homogenous assay-linked immunosorbent assay (AlphaLISA) is comprised of a bead-based immunoassay that is used for small molecule detection. In this study, a novel AlphaLISA was developed and optimized for the detection of Shiga-toxin 2 (Stx2). Efficacy and sensitivity trials showed the AlphaLISA could detect ≥0.5 ng/mL of purified Stx2, which was comparable to the industry-standard enzyme-linked immunosorbent assay (ELISA) tests for Stx2 detection. In addition, evaluation of Shiga toxin-producing *Escherichia coli* (STEC)-inoculated Romaine lettuce and ground beef samples demonstrated that both the AlphaLISA and the ELISA were able to discern uninoculated samples from 1× and 10× diluted samples containing ~10 CFU/mL of STEC enriched in modified tryptic soy broth with mitomycin C for 16 h. Overall, the increased signal-to-noise ratios indicated a more robust signal was produced by the AlphaLISA compared to the ELISA and the delineation of higher toxin concentrations without the need for sample dilution implied a greater dynamic range for the AlphaLISA. Implementation of the newly developed AlphaLISA will allow for more rapid analysis for Stx2 with less manual manipulation, thus improving assay throughput and the ability to automate sample screening while maintaining detection limits of 0.5 ng/mL.

## 1. Introduction

Food poisoning due to Shiga toxin-producing *Escherichia coli* (STEC) is a consistent cause for concern in the United States because of its association with hemorrhagic colitis. Clinically, hemorrhagic colitis is characterized by the onset of a variety of symptoms including nausea, vomiting, abdominal pain, diarrhea, and bloody stools with approximately 5% of cases progressing to a more severe form of clinical disease known as hemolytic uremic syndrome (HUS) [1]. Expenses related to STEC illnesses are difficult to quantify; however, estimates from summary health measures such as the annual number of illnesses, hospitalizations, and deaths as estimated by the Centers for Disease Control and Prevention (CDC) [2] can be used to approximate the costs to be around $300 M and a loss of 1700 quality-adjusted life years annually [3].

Rapid and accurate identification of STEC is imperative for the protection of several facets of human health. For example, stopping STEC contamination in food production is critical for curtailing the number of infections and preventing full-scale outbreaks from occurring, while obtaining an accurate diagnosis upon infection is crucial for ensuring proper care for patients. Because treatment of STEC infections with antibiotics greatly increases the risk of serious complications resulting from the development of the condition known as hemolytic uremic syndrome (HUS) [4,5] and Shiga toxin is known to be essential for the development of HUS from STEC infections [6]; the production and subsequent release of Shiga toxin has been a topic of intense investigation. Production of Shiga toxin is both a distinguishing feature and an important virulence factor for STEC strains. It can be expressed by strains carrying the *stx* gene, which is encoded for by a lambdoid bacteriophage [7,8]. Current research separates Shiga toxin into two main antigenically distinct groups, Shiga toxin 1 (Stx1) and Shiga toxin 2 (Stx2) [9]. Both toxins consist of a single A-subunit of ~32 kDa and five B-subunits of ~7.7 kDa each [10]. However, marked differences in the toxicity level exist between the two groups. For example, Stx2 types have an LD50 approximately 400 times lower than Stx1 [11] despite having ~60% identity at the amino acid level between the two groups [10]. Each of the two main groups can be further subdivided into the following variants: Stx1a, Stx1c, Stx1d, and Stx2a, Stx2b, Stx2c, Stx2d, Stx2e, Stx2f, and Stx2g [12]. The Food Safety and Inspection Service, an agency of the United States Department of Agriculture, has undertaken routine screening of meat samples for the presence of *stx* in order to identify the presence/absence of STEC in the food supply chain [13,14].

Because culture-based STEC detection can be long and laborious, alternative methods for identifying strains that produce Shiga toxin have emerged. More rapid antibody-based detection tests, specifically enzyme-linked immunosorbent assays (ELISAs) [15,16], antibody-based lateral flow assays (LATs) [17], and immunomagnetic separation assays (IMS) [18,19] have been employed as common testing platforms for the detection of STECs and Shiga toxin in lieu of traditional microbiological detection methods such as colony plating on selective media. A new platform known as the AlphaLISA, or amplified luminescent proximity homogenous assay-linked immunosorbent assay, utilizes both bead- and antibody-based technologies for pathogen or toxin detection. In this system, two different antibody-coated beads (aka. “donor” and “acceptor” beads) are used to bind either the same antigenic region on a target or two distinct antigenic regions of a target that are within the vicinity of one another. The donor beads contain a photosensitizing agent, which upon laser excitation at 680 nm, causes the donor bead to emit ~60,000 singlet oxygen molecules per second [20]. These singlet oxygen molecules then react with thioxene derivatives within the acceptor beads, resulting in the production of a chemiluminescent signal at 340–350 nm [21]. This signal activates fluorophores, also contained within the acceptor beads, resulting in a detectable signal consisting of emitted fluorescence with a narrow bandwidth centered around 615 nm. Because the half-life of the singlet oxygen is 4 microseconds, its diffusion distance is limited to approximately 200 nm in aqueous solutions. Thus, if the beads are not in close enough proximity, the singlet oxygen molecules from the donor bead decay to their ground state prior to reaching the acceptor beads and no signal is emitted.

The AlphaLISA has many advantages over the ELISA; namely its high sensitivity, relatively quick testing time, reduced hands-on workflow resulting from the ability to sequentially overlay the reagents, and it is easily adaptable to automation and high-throughput screening [22]. In this study, the first AlphaLISA for the detection of a bacterial toxin in foods was developed using the presence of Shiga toxin 2 (Stx2) as a marker for STEC contamination in foods. Comparisons were made between the newly developed AlphaLISA and an industry-standard ELISA in two different food matrices, Romaine lettuce and ground beef, to further evaluate the assay’s utility as a rapid detection method.

## 2. Results

### 2.1. Antibody Configuration, Titration, and Gain Settings for Amplified Luminescent Proximity Homogenous Assay-Linked Immunosorbant Assay (AlphaLISA)

**Figure 1:**
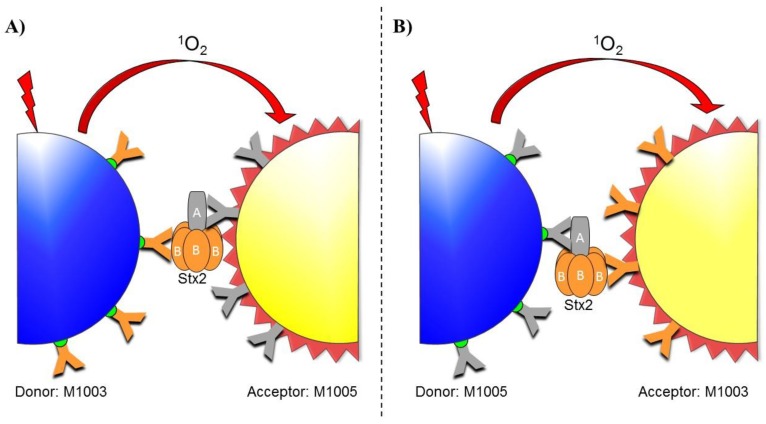
Schematic representation of the sandwich amplified luminescent proximity homogenous assay-linked immunosorbent assay (AlphaLISA) for Shiga toxin detection. Two sets of monoclonal antibodies were employed in this assay (orange and gray Y’s): one specific for the (**A**) subunit (M-1005) and other for the (**B**) subunit (M-1003) of Stx2. One set of antibodies was biotinylated (green hemisphere) to allow association with streptavidin-coated donor beads (blue sphere), while the other antibody set was directly conjugated to the acceptor beads (yellow sphere). When Stx2 is present, the donor and acceptor beads are colocalized through binding of the antibodies to the different subunits of the common antigen. Upon excitation by an Alpha laser at 680 nm (lightning bolt), the donor beads emit singlet oxygen molecules that react with the acceptor beads. Ultimately, energy transfer results in the emission of light by the acceptor beads, thus creating a detectable fluorescence signal at 615 nm. The following two formats were analyzed during assay development: (**A**) Antibody M-1003 was biotinylated and thus associated with the donor beads while M-1005 was conjugated to the acceptor beads and (**B**) antibody M-1005 was biotinylated and thus associated with the donor beads while M-1003 was conjugated to the acceptor beads.

**Figure 2:**
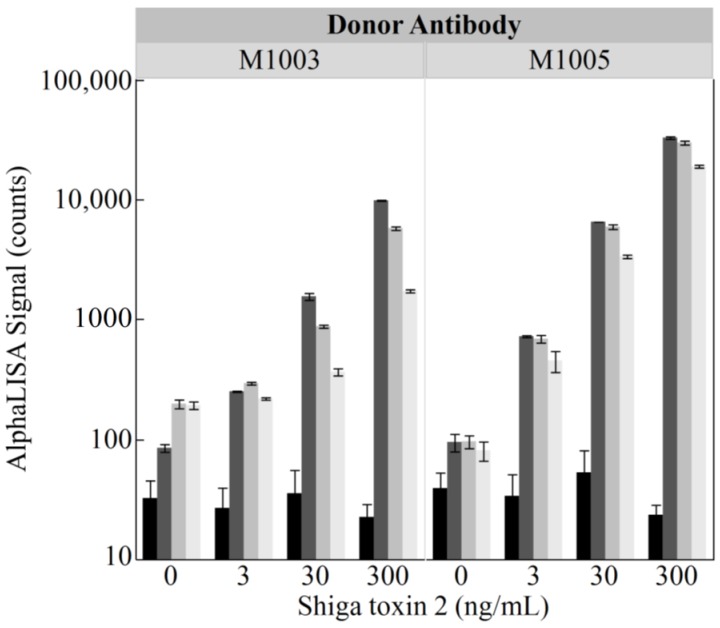
Optimization of donor/acceptor bead pairing and antibody concentration. A multifactorial test involving the donor/acceptor bead pairing, donor antibody concentration, and Stx2 concentration was performed to identify the optimal assay parameters for the AlphaLISA. AlphaLISA signals were recorded for the following factors: (a) bead pairings consisting of donor M-1003 with 25 µg acceptor M-1005 (left panel) versus donor M-1005 with 25 µg acceptor M-1003 (right panel); (b) donor antibody concentrations of 0 (black bars), 0.3 (dark gray bars), 1.0 (medium gray bars), and 3.0 nM (light gray bars); and (c) Stx2 concentrations of 0, 3 ng/mL, 30 ng/mL, and 300 ng/mL. Emitted light was measured on a BioTek Cytation 5 in Alpha mode at a gain setting of 100. Bars represent the mean values (*n* = 4) with error bars denoting the standard deviation.

The AlphaLISA design presented here (Figure 1) utilizes two different monoclonal antibodies with specific affinity to Stx2 for incorporation onto the donor and acceptor beads. Antibody M-1005 binds the Shiga toxin 2 A-subunit while M-1003 binds the Shiga toxin 2 B-subunits. These antibodies were chosen so that the two beads would bind to different areas of the toxin, as antibodies with the same target site may compete with each other rather than trap the toxin between them. During assay development, antibody M-1003 was first used as the biotinylated antibody for association with the streptavidin-coated donor bead while the M-1005 antibody was conjugated to the acceptor bead (Figure 1A). Then, antibody M-1003 was conjugated to the acceptor bead while the M-1005 antibody was used as the biotinylated antibody for association with the streptavidin-coated donor bead (Figure 1B). In addition to testing both antibody configurations, the final concentration of the biotinylated antibody was also varied (0, 0.3, 1.0, and 3.0 nM) while keeping the acceptor-conjugated antibody beads constant to ensure optimal performance would be achieved for the detection of Stx2 in the AlphaLISA format. Results of this assay (Figure 2) showed that using 0.3 nM of biotinylated M-1005 antibody for association with the donor, while conjugating antibody M-1003 to the acceptor produced the largest signal intensities. Furthermore, this condition demonstrated the ability to differentiate between all levels of Stx2 as the *p*-values of independent Student’s *t*-tests were less than 0.003. Since the response for this condition displayed the largest signal amplitudes and the ability to differentiate between each level of Stx2 tested, this configuration was subsequently used in the remainder of the assays presented. The final parameter examined in an effort to maximize the sensitivity and dynamic range of the AlphaLISA was the gain setting for the photomultiplier tube on the plate reader. A multifactorial test was performed using 0.3 nM of biotinylated M-1005 as the donor and conjugating M-1003 to the acceptor bead in an assay evaluating four levels of Stx2 (0, 3, 30, and 300 ng/mL) at three different gain settings (gain = 100, 125, and 150). While we observed larger signal intensities with the higher gains, we also noted that for some conditions, the assay was unable to differentiate between levels of Stx2 using a Student’s *t*-test with an alpha value of 0.05. From this, it was determined that a gain setting of 100 was optimal because it demonstrated the greatest precision and was the best at resolving the lower levels of toxin (data not shown).

### 2.2. Sensitivity of the AlphaLISA Compared to the Enzyme-Linked Immunosorbent Assay (ELISA)

**Figure 3:**
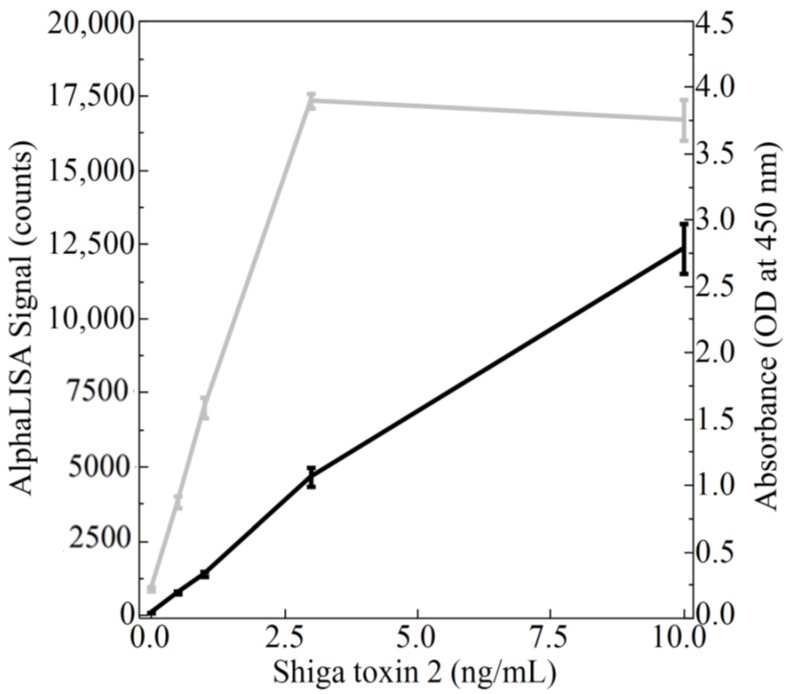
Detection of purified Stx2 using the AlphaLISA and the enzyme-linked immunosorbent assay (ELISA). Varying concentrations of purified Stx2 ranging from 0–10 ng/mL were analyzed using both the AlphaLISA (black line) and the ELISA (gray line). The response from both assays were quantified on a BioTek Cytation 5 with the emitted light from the AlphaLISA measured via the Alpha mode at a gain setting of 100 (left *y*-axis) and the absorbance values of the ELISA measured at 450 nm (right *y*-axis). Mean values from 6 trials were plotted with error bars denoting the standard deviation.

Using the optimized parameters, purified Stx2 was then used to determine the sensitivity for the newly developed AlphaLISA. Varying concentrations of Stx2 (0, 0.5, 1, 3, and 10 ng/mL) were assayed using the AlphaLISA and the results recorded (Figure 3 black line). To benchmark the newly developed AlphaLISA against similar technologies, the same concentrations of Stx2 were also assayed using a commercially available ELISA (Figure 3 gray line). Six independent trials for each assay were conducted and the results were analyzed via independent Student’s *t*-tests with alpha <0.05. The analysis demonstrated the ability of both the AlphaLISA and the ELISA to differentiate samples that do not contain Stx2 from samples containing 0.5, 1, 3, and 10 ng/mL of Stx2. In addition, independent *t*-tests (alpha < 0.05) demonstrated the ability of the assay to differentiate between all levels of Stx2 concentrations. Although the signal for both the AlphaLISA and the ELISA increased as the concentration of Stx2 increased, it is worth noting that the signal for the ELISA appeared to reach the maximum absorbance at 3 ng/mL of Stx2. (Note the maximum absorbance signal measurable by the instrument is 4.0.) The AlphaLISA, however, continued to show a marked increase in signal from 3 to 10 ng/mL.

### 2.3. Detection of Stx2 in Food Matrices Using the AlphaLISA

**Figure 4:**
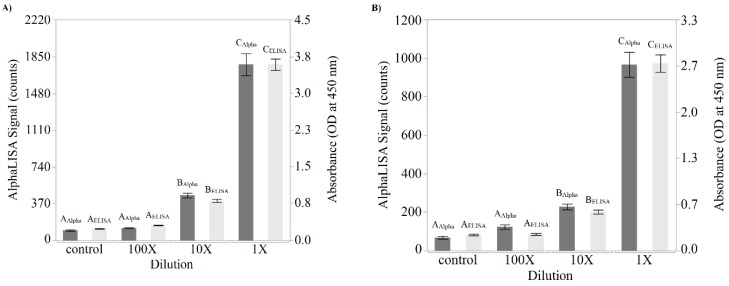
Detection of Stx2 in Shiga toxin-producing *Escherichia coli* (STEC)-inoculated foods using the AlphaLISA and ELISA. Stx2 production was induced using mitomycin C in STEC-inoculated (**A**) Romaine lettuce and (**B**) ground beef. Undiluted (1×) or diluted (10× and 100×) samples were subsequently assayed for the presence of Stx2 using both the AlphaLISA (dark gray bars) and the ELISA (light gray bars). Control samples contained uninoculated lettuce and ground beef samples, respectively. Responses were quantified on a BioTek Cytation 5 with the emitted light from the AlphaLISA measured via Alpha mode at a gain setting of 100 (left *y*-axis) and the absorbance values of the ELISA measured at 450 nm (right *y*-axis). Mean values from 3 independent trials containing 2 replicates were plotted with error bars denoting the standard deviation. Significance between AlphaLISA signal or absorbance values are denoted by dissimilar letters as determined by a Student’s *t*-test at a 95% confidence level.

In addition to the testing performed on purified toxin, the ability of the AlphaLISA to detect the presence of Stx2 in inoculated lettuce and ground beef samples was also examined to ascertain the functionality of the AlphaLISA in food matrices. These two food matrices were chosen because of their previous implication in STEC outbreaks [23,24,25,26]. For comparison purposes, lettuce inoculated with 9 CFU/mL of a Shiga toxin 2-producing *E. coli* O145 strain was tested by both AlphaLISA and ELISA for the presence of Stx2. During an overnight incubation, expression of Stx2 was induced in samples through the addition of mitomycin C. Samples consisting of 1× (undiluted) material, a 10× dilution, or a 100× dilution were then assayed (Figure 4A). Lettuce samples that had not been inoculated with STEC but were incubated in the same broth and under the same conditions were used as controls for this study. Because the output of the ELISA and the AlphaLISA differ, with the ELISA being a colorimetric assay measuring absorbance while the AlphaLISA yields emitted fluorescence, the numeric values are not directly comparable. However, both assays demonstrate a dose-dependent response. Both the AlphaLISA and ELISA were repeated 3 times in duplicate, and the data was analyzed using Student’s *t*-tests, alpha = 0.05. In both the AlphaLISA and the ELISA, the 1× lysate dilution, the 10× lysate dilution and the control, were significantly different (*p* < 0.0001). However, the 100× lysate dilution could not be differentiated from the uninoculated control in either the AlphaLISA or the ELISA as *p* = 0.73 and 0.34, respectively. This implies that the AlphaLISA and the ELISA displayed similar Stx2 detection capabilities in lettuce.

Testing of the inoculated ground beef samples was performed in a similar fashion to the lettuce samples using both the AlphaLISA and the ELISA to validate the presence of Stx2 (Figure 4B). The resulting data was also similar with both the AlphaLISA and the ELISA demonstrating a significant difference between the 1× lysate dilution, the 10× lysate dilution and the control (*p* < 0.0001). However, the 100× lysate dilution could not be differentiated from the uninoculated control in either the AlphaLISA or the ELISA as *p* = 0.18 and 0.91, respectively. Taken together, the data from both the lettuce and the ground beef illustrate that the AlphaLISA is comparable to the ELISA in terms of sensitivity and the ability to detect the presence of Stx2 in inoculated food matrices.

### 2.4. AlphaLISA Sensitivity Using Increased Reagent Volumes

**Figure 5:**
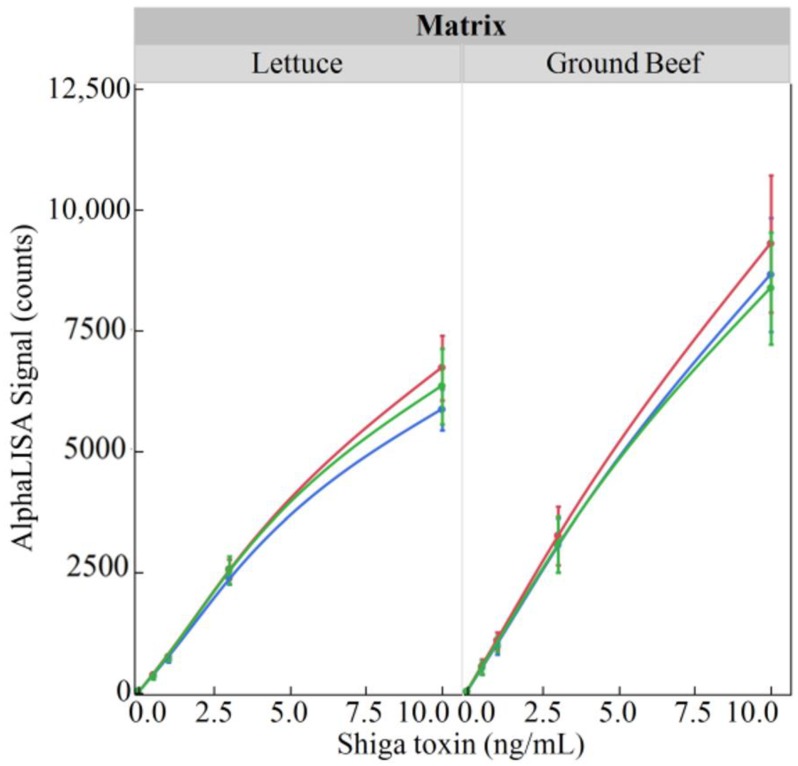
The effect of increasing reaction components on the sensitivity of the AlphaLISA for Stx2 in food matrices. Purified Stx2 (0, 0.5, 1.0, 3.0 or 5.0 ng/mL) was added to uninoculated lettuce (**left**) panel or ground beef (**right**) panel and the effect of increasing all of the reaction components (1×-blue line, 2×-red line, or 3×-green line) in the AlphaLISA signal was determined. Responses were quantified on a BioTek Cytation 5 with the emitted light from the AlphaLISA measured via Alpha mode at a gain setting of 100. Mean values from 2 independent trials, each containing 2 replicates are plotted with error bars denoting the standard deviation.

In an attempt to enhance the sensitivity of the AlphaLISA, a series of experiments were conducted using increased reagent volumes for the AlphaLISA (Figure 5). The premise behind these experiments was that the small sample volume of the AlphaLISA (5 µL) could be the limiting factor with regards to the sensitivity of the overall assay. Therefore, by simply increasing all assay components, the limit of detection may also increase accordingly. The ability of the AlphaLISA to detect 0, 0.5, 1.0, 3.0, or 10.0 ng/mL of purified Stx2 in the presence of a food matrix (lettuce or ground beef) using either the original assay components (1×), doubling the assay components (2×), or tripling the assay components (3×) was determined. From this, it was shown that when the assay components were increased from 1× to 2× that a corresponding increase in AlphaLISA signal was also detected in both lettuce and ground beef. However, the results were not consistent when assay components were increased to 3×. When the components of the assay were tripled, the AlphaLISA signal was higher than that seen for the 1× but not the 2× amounts in the lettuce; whereas this level resulted in the lowest signal in ground beef when compared to both the 1× and 2× assays. 

## 3. Discussion

AlphaLISA is a versatile technology that employs oxygen-channeling chemistry [27,28] and has been used for the detection of a wide variety of analytes from proteins to peptides to other small molecules. Currently, hundreds of different AlphaLISA biomarker detection kits are commercially available ranging in application from medicine (for tracing unwanted host cell proteins during industrial scale production of biotherapeutics), to agriculture (for the detection of aflatoxins in foods and animal feed), to basic research (as an alternative to the electrophoretic mobility shift assay for the detection of DNA-protein interactions). The AlphaLISA is considered a “no wash” alternative to the ELISA because it does not require any wash or separation steps [22]. The reagents are simply overlaid in succession, which greatly simplifies the protocol and makes the AlphaLISA highly amendable to high-throughput automated screening. Because of this, the AlphaLISA would be the superior assay when a considerable number of samples are to be analyzed under a standard operating procedure, such as is the case with the assessment of food quality and safety around the globe. Here, an AlphaLISA was developed to identify the presence of foodborne pathogens through the detection of Shiga toxin 2 in food samples. 

Direct comparisons between the newly developed AlphaLISA and the ELISA were performed using both purified Stx2 and STEC-inoculated Romaine lettuce and ground beef samples. The suggestion provided by Perkin Elmer to estimate the limit of detection is to add 3 standard deviations to the mean of the “zero analyte” condition. Using this suggestion and the data presented in Figure 2, Table 1 was generated. Although the limits of detection were only slightly better for the AlphaLISA compared to the ELISA in terms of toxin concentration (0.10 ng/mL versus 0.14 ng/mL, respectively), when one considers that the ELISA employs a 100 µL sample while the AlphaLISA employs a 5 µL sample, quantitation of the amount of toxin on a per weight basis demonstrated that the AlphaLISA is the superior assay (0.5 pg for the AlphaLISA versus 14.3 pg for the ELISA). Interestingly, an attempt was made to increase the sensitivity of the AlphaLISA by doubling and tripling the components in the reaction including the amount of sample per assay (Figure 5). Although a corresponding increase in signal was detected when the reaction components were doubled (1× to 2× comparison), a tripling of the reaction components showed a decrease in signal intensity (2× to 3× comparison). The reason behind this deleterious effect with regard to signal production upon tripling of the reaction components is unknown and was not further pursued since the mean signal produced by the 1×, 2×, and 3× component mixtures were not statistically different.

The AlphaLISA is also superior because ELISAs typically have a narrower dynamic range, thus requiring the testing of multiple sample dilutions to accurately measure antigen concentration. As can be seen from Figure 3, samples within the range of 0.5 ng/mL to 3 ng/mL were detectable by the ELISA while samples containing 10 ng/mL of Stx2 fell outside of the absorbance range of the Cytation 5 microtiter plate reader, and thus could not be accurately measured unless diluted. Conversely, the AlphaLISA was able to measure concentrations within the 10 ng/mL range and possibly higher without dilution given the fact that AlphaLISA counts of ~1,600,000 have been recorded at higher gains using the Cytation 5 by Biotek (data not shown). Given that the saturation point was reached for ELISA but not for the AlphaLISA suggests that the dynamic range of the AlphaLISA is greater than that of the ELISA. While the observation of a broader dynamic range is consistent with what has been previously reported [22], larger scale studies would need to be conducted to precisely define the dynamic range of the assays. Regardless, the signal-to-noise ratio for the AlphaLISA was much larger than that of the ELISA (Table 1), indicating a better specification since there is more desired signal compared to background noise.

Antibody selection is crucial for the development of a functional AlphaLISA, including the fact that the specificity of the antibodies ultimately determines the accuracy of selection for the assay. The design presented here is specific for Stx2 (the more toxic of the two Stx types [11]) because it utilizes two different monoclonal antibodies with the donor antibody M-1005 specifically recognizing the Stx2 A-subunit and the acceptor M-1003 specifically recognizing the Stx2 B-subunit. Although the determination of which antibody was selected as the donor compared to the acceptor was done empirically, it has been hypothesized that this configuration was the most robust based on the following. First, the selection of two different antibodies ensures that the binding reaction can proceed without interference from the antibodies competing for binding sites, and allows the binding reaction to be more efficient compared to assays which utilize either the same antibody or antibodies that recognize different peptides within the same protein. Second, the AlphaLISA signaling cascade allows a single donor to activate multiple acceptors, and thus the creation of a more intense output signal compared to that of only a single acceptor in the presence of multiple donors. Because Stx2 consists of an A subunit noncovalently joined to a pentamer of identical B subunits, there are theoretically five times the number of acceptor particles surrounding every donor particle in the assay. Taken together, the configuration shown in Figure 1B would likely emit the most intense signal because the single A subunit donor could emit to multiple B subunit acceptors, which corresponded to the signal intensity observed experimentally. This result was analogous to what was seen for the detection of *Bacillus anthracis* spores via an AlphaLISA [29].

Ultimately, we developed a novel AlphaLISA for the detection of Stx2 and demonstrated its ability to identify the presence of Stx2 not only in phosphate buffered saline (PBS) but in two food matrices as well. Because the AlphaLISA can be performed in ~1.5 h (compared to the ~2.5 h needed for the ELISA), does not require the plate manipulation needed to wash the sample wells like the ELISA, and appears to have a larger dynamic range; the AlphaLISA is the superior method for the detection of Stx2 when large numbers of samples are to be queried. Other potential benefits may also be realized such as the fact that the AlphaLISA requires the toxin to be intact since the signal is dependent upon the A and B subunits being within a set distance to one another. This AlphaLISA might also be applicable for quantifying the amount of Stx2 in other matrices, indicating a use not only for detecting the presence of STECs in food as presented here but for use in clinical samples consisting of serum/stool [30,31] or for research investigating variables related to *stx* induction as well [32].

## 4. Materials and Methods

### 4.1. Preparation of Antibodies

Antibodies known to detect a majority of the Stx2 subtypes were selected from purified Abraxis (Warminster, PA, USA) in-house stocks stored in PBS (Abraxis LLC). The monoclonal antibody M-1003 binds the Shiga toxin B-subunits, while the monoclonal antibody M-1005 binds the Shiga toxin A-subunit. These two antibody stocks were also biotinylated using the Thermo Fisher Scientific (Waltham, MA, USA) EZ-Link Sulfo-NHS-LC-LC-Biotin (prod. #21338) according to manufacturer instructions, with a one-hour incubation at room temperature followed by removal of excess biotin with a Zeba Spin Desalting Column (Thermo Fisher Scientific prod. #89893). Biotinylated antibodies were stored in PBS at −20 °C. Antibody concentrations for both the biotinylated and stock antibodies were measured at 280 nm using a DeNovix microspectrophotometer (DeNovix Inc., Wilmington, DE, USA).

### 4.2. Preparation of AlphaLISA Donor and Acceptor Beads

Uncoupled AlphaLISA donor and acceptor beads were purchased from PerkinElmer, Inc. (Waltham, MA, USA; acceptor prod. #6772001 and donor prod. #6760002S). The streptavidin-coated donor beads did not need additional modification; however, the acceptor beads required antibody coupling prior to the start of the AlphaLISA assay. Two sets of acceptor beads were prepared (M-1003 and M-1005) using a modified version of the procedures found in the PerkinElmer “ELISA to Alpha Immunoassay Conversion Guide,” section “Protocol for Direct Conjugation of an Antibody to an AlphaLISA Acceptor Bead” [33]. Briefly, two sets of 25 µL of 20 mg/mL acceptor beads (equaling 0.5 mg each) were washed with 25 µL of PBS in 1.5 mL microfuge tubes, centrifuged at 16,000× *g* for 15 min (Eppendorf centrifuge), and the supernatants discarded. To each set of 0.5 mg beads, 0.05 mg of either M-1003 or M-1005 antibody (non-biotinylated), 0.625 µL of 10% Tween-20 (Sigma-Aldrich, St. Louis, MO, USA; prod. #P7949), and 5 µL of 25 mg/mL NaBH_3_CN (Acros Organics, Thermo Fisher Scientific; prod #168550100) was added and brought to a final volume of 100 µL with 130 mM sodium phosphate (Sigma-Aldrich prod. #S0876/0). The bead pellet was resuspended in this mixture by pipetting, and incubated statically for 18 h at 37 °C. Blocking of unreacted sites was then performed by adding 5 µL of 65 mg/mL carboxy-methoxylamine (CMO) (Sigma-Aldrich prod. #C13408) in 0.8 M sodium hydroxide (Alfa Aesar, Haverhill, MA, USA; prod. #35631) to the previous reactions, and incubating for 1 h at 37 °C with an occasional gentle vortex. Finally, the beads underwent a series of wash steps as follows. The two conjugated, blocked bead solutions were centrifuged at 16,000× *g* for 15 min and the supernatants were discarded. The pellets were resuspended in 100 µL of 0.1 M Tris-HCl pH 8.0 (Sigma-Aldrich prod. #T3253) and centrifuged again at 16,000× *g* for 15 min. The previous step was repeated twice more, and the beads were resuspended by pipetting up and down in 100 µL of PBS with 0.05% ProClin-300 (Sigma-Aldrich prod. #48914) added as a preservative, for a final concentration of 5 mg/mL. The two separate antibody-conjugated acceptor bead solutions were vortexed and sonicated with twenty 1-s pulses in a water bath sonicator and stored at 4 °C in the dark.

### 4.3. Preparation of Shiga Toxin 2 Standards

Shiga toxin 2 was purchased from Toxin Technology (Sarasota, FL, USA, prod. STX-2), at 0.5 mg/mL, and diluted to 3, 30, and 300 ng/mL in AlphaLISA buffer (25 mM HEPES pH 7.4 (VWR International, Radnor, PA, USA; prod. #0511) containing the following from Sigma-Aldrich 0.5% Triton-X 100, 0.1% casein (prod. #C-5890), 1 mg/mL dextran (prod. #31390), and 0.05% ProClin-300prod. #48914). 

### 4.4. AlphaLISA Antibody Pair and Concentration Optimization

Once the reagents (biotinylated antibodies, antibody-conjugated acceptor beads, and donor beads) and Shiga toxin 2 standards were prepared, AlphaLISA optimization began. Stx2 standards at 0, 3, 30, and 300 ng/mL in AlphaLISA buffer were evaluated to determine optimal antibody pairing and titer level. As per the recommended PerkinElmer procedures in the ELISA to Alpha Immunoassay Conversion Guide, 5 µL of Stx2 standards were added to 1/2-area plate (Perkin Elmer prod. #6005560) wells. Then, 10 µL of biotinylated M-1003 or M-1005 antibody (diluted to a final concentration of 0, 0.3, 1, or 3 nM in a 50 µL total reaction volume) was added, followed by 10 µL of M-1003 or M-1005 antibody-conjugated acceptor beads (diluted to a final concentration of 10 µg/mL in 50 µL total reaction volume). M-1003-conjugated acceptor beads were only added to wells that had biotinylated-M-1005 added, and M-1005-conjuated acceptor beads were only added to wells that had biotinylated-M-1003 added in order to have complementary antibody pairs instead of competing pairs. The plate was incubated at room temperature for 1 h. Next, 25 µL of streptavidin-coated donor beads were added (for a final concentration of 40 µg/mL in 50 µL total reaction volume) under low light conditions to prevent photobleaching of the beads. This results in a final 50 µL total reaction volume. The plate was incubated in the dark at room temperature for 30 min. Total in-assay workflow time was approximately 1 h and 45 min. Microtiter plates were then read using a BioTek Cytation 5 (BioTek Instruments, Inc., Winooski, VT, USA) in Alpha measurement mode with a gain setting of 100. 

### 4.5. Analysis of Purified Shiga Toxin 2

Shiga toxin 2 standards were prepared as described above. Samples consisting of 5 µL of the 0, 0.5, 3, 30, and 300 ng/mL Stx2 standards were assayed via the AlphaLISA using only the 0.3 nM of biotinylated M-1005 antibody for association with the donor and the acceptor-conjugated M-1003 antibody as described above. ELISA testing was conducted simultaneously using the Abraxis Shiga Toxin 2 ELISA kit (Abraxis prod. #542010) using 100 µL of the 0, 0.5, 3, 30, and 300 ng/mL Stx2 standards following the manufacturer’s instructions. Absorbencies for the ELISA were also measured on the BioTek Cytation 5 at 450 nm five min after the addition of stop solution.

### 4.6. Preparation of Lettuce and Ground Beef Samples

Lettuce and ground beef samples were prepared following the procedures described previously [15]. Briefly, Romaine lettuce was purchased from a local grocery store, the outermost leaves were removed, while the remaining leaves were chopped into ~1 cm^2^ pieces on a 70% ethanol sterilized surface. Ground beef was also purchased from a local grocery store, separated into 25 g quantities, and kept frozen until used. STEC inoculum was prepared from a frozen *E. coli* O145:H28 bacterial stock by growing a small chip of frozen stock in Tryptic Soy Broth (TSB) (Becton Dickinson, East Rutherford, NJ, USA; prod. #211825) overnight at 37 °C shaking at 120 rpm in a 15 mL Corning Falcon tube. The following day, the overnight culture was serially diluted in glass dilution tubes with peptone water (Becton Dickinson prod. #218105), and stored at 4 °C. The number of colony forming units (CFU) present were determined by plating 1 mL of each dilution onto a Tryptic Soy Agar (TSA) plate (Becton Dickinson prod. #236950), which was incubated at 37 °C overnight. Inoculum levels were 9 and 10 CFU/mL for the lettuce and beef samples, respectively.

Samples were incubated in broth at a 1:4 ratio as follows. Lettuce or ground beef partitioned into 25 g samples was placed into filter Stomacher bags (Seward Laboratory Systems Inc., Islandia, NY, USA). One milliliter of ~10 CFU/mL STEC inoculum was added to the samples, or 1 mL of peptone water for the uninoculated negative control samples. Next, 75 mL of modified mTSB (TSB with 10 g/L Casamino Acids (Neogen Corp., Lansing, MI, USA; prod.# 7229A) and 66.7 ng/L mitomycin C (Sigma-Aldrich #M-0503) antibiotic toxin inducer), pre-warmed to 42 °C, was measured into all samples and hand-massaged to mix well and break up any clumps. Samples were subsequently incubated overnight (16 h) statically at 42 °C. The following day, 10 mL samples were removed from the outer portion of the Stomacher bags and stored at −80 °C in 15 mL Falcon tubes till use. Dilutions of lettuce and ground beef samples were made in AlphaLISA buffer in a similar fashion to that of the Shiga toxin 2 standards.

### 4.7. AlphaLISA and ELISA Comparison Food Matrices

Testing of the inoculated lettuce and ground beef samples described above was performed as follows. For the AlphaLISA, 5 µL of either inoculated or uninoculated control samples were added to Perkin Elmer 1/2-area plates, and tests were performed as detailed in the AlphaLISA optimization section above using the optimized conditions of 0.3 nM biotinylated-M-1005 antibodies with M-1003-conjugated acceptor beads. ELISA testing was conducted simultaneously using the Abraxis Shiga Toxin 2 ELISA kit using 100 µL of sample and following the manufacturer’s instructions. Microtiter plates were read on a BioTek Cytation 5 for either the fluorescence signal using the Alpha mode (gain setting of 100) for the AlphaLISA or for absorbance at 450 nm five minutes after the addition of stop solution for the ELISA.

### 4.8. AlphaLISA Using 1×, 2× and 3× Components

Stx2 samples containing 0, 0.5, 1.0, 3.0, and 10.0 ng/mL of the Stx2 standards were prepared in uninoculated lettuce as described above. From there 5, 10 or 15 µL of the Stx2 containing lettuce was assayed using 10, 20, or 30 µL of prepared biotinylated antibody and 10, 20, or 30 µL of the prepared acceptor-conjugated beads for the 1×, 2×, and 3× experiments, respectively. (The AlphaLISA beads for all assays consisted of the 0.3 nM of biotinylated M-1005 antibody for association with the donor- and the acceptor-conjugated M-1003 antibody.) The plate was incubated at room temperature for 1 h before 25, 50, or 75 µL of streptavidin-coated donor beads were added under low light conditions resulting in total reaction volumes of 50 µL (1×), 100 µL (2×), or 150 µL (3×). Microtiter plates were incubated as before with the resulting signal quantified on the BioTek Cytation 5 as previously described. Identical assays were performed except uninoculated ground beef was used as the matrix instead of lettuce for these studies. 

## Figures and Tables

**Table 1:** Comparisons of the limit of detection and signal-to-noise ratios of the AlphaLISA versus the ELISA.

Assay	Average Zero Signal	3× SD	LOD (ng/mL)	Signal-to-Noise (S:N) ^a^			
				0.5 ng/mL	1 ng/mL	3 ng/mL	10 ng/mL
AlphaLISA	75.92	63.285	0.100	10	18	61	163
ELISA	0.21767	0.1665	0.143	4	7	18	17

SD = standard deviation, LOD = limit of detection. ^a^ Signal-to-noise ratios were calculated by dividing the signal mean of the stated assay upon the addition of either 0.5, 1, 3, or 10 ng/mL purified Stx2 to phosphate buffered saline (PBS) by the signal mean in samples with no Stx added to PBS.
